# Knocking out the LRRK2 gene increases sensitivity to wavelength information in rats

**DOI:** 10.1038/s41598-024-55350-9

**Published:** 2024-02-29

**Authors:** Freja Gam Østergaard

**Affiliations:** 1 H. Lundbeck A/S, Ottiliavej 9, 2500 Valby, Denmark; 2 Present Address: GELIFES, University of Groningen, Nijenborgh 7, 9747 AG Groningen, The Netherlands

**Keywords:** Parkinson's disease, Colour vision, Predictive markers

## Abstract

Leucine-rich repeat kinase 2 (LRRK2) is a gene related to familial Parkinson’s disease (PD). It has been associated with nonmotor symptoms such as disturbances in the visual system affecting colour discrimination and contrast sensitivity. This study examined how deficiency of LRRK2 impacts visual processing in adult rats. Additionally, we investigated whether these changes can be modelled in wild-type rats by administering the LRRK2 inhibitor PFE360. Visual evoked potentials (VEPs) and steady-state visual evoked potentials (SSVEPs) were recorded in the visual cortex and superior colliculus of female LRRK2-knockout and wild-type rats to study how the innate absence of LRRK2 changes visual processing. Exposing the animals to stimulation at five different wavelengths revealed an interaction between genotype and the response to stimulation at different wavelengths. Differences in VEP amplitudes and latencies were robust and barely impacted by the presence of the LRRK2 inhibitor PFE360, suggesting a developmental effect. Taken together, these results indicate that alterations in visual processing were related to developmental deficiency of LRRK2 and not acute deficiency of LRRK2, indicating a role of LRRK2 in the functional development of the visual system and synaptic transmission.

## Introduction

The *leucine-rich repeat kinase 2* (*LRRK2*) gene, formerly referred to as the *PARK8* gene due to its association with Parkinson’s disease (PD)^1^*,* encodes a large protein expressed throughout the body^2^, especially in the lungs, kidneys and immune cells^3^. LRRK2 contains several different domains for protein‒protein interactions. It is present in the cytoplasm bound to 14-3-3 and at cell membranes, forming a homodimer^4^. Functionally, LRRK2 interacts with multiple Rab proteins^5–7^, which in turn acts as regulators of membrane trafficking^4^. In neurons, LRRK2 has been associated with synaptic vesicle endocytosis^6^ and autophagy^8^. In line with its association with PD, LRRK2 seems to localize to dopaminoceptive areas^1^. In addition to the PD-driven association with dopamine, both gain-of-function models^9^ and knock-in models^10^ of LRRK2 has shown changes in glutamatergic activity when compared to wildtype animals. LRRK2 may also have a more widespread role in neurodevelopment, as it localizes to the ventricular and subventricular zones of the embryonic telencephalon in mice^2^.

Most research on this topic has focused on gain-of-function mutations in LRRK2^4^, as this is the most common type of mutation found in PD^11^. Studies carried out in LRRK2-KO animals have generally been used to examine the risks of LRRK2 inhibition, as this could be a potential therapeutic approach for PD^11^. In addition to genetic models of LRRK2 deficiency, models of pharmacological modulation have been investigated using an LRRK2 inhibitor called PFE360, which targets the kinase activity of LRRK2. It should however be noted that PFE360 has numerous potential off-target effects^12^. Chronic inhibition of LRRK2 has been shown to cause accumulation of lipids in the kidneys^11,13^. Administering PFE360 in the context of PD has been shown to normalize various phenotypical traits such as aberrant neuronal firning^14^.

Previous studies in both humans and flies have indicated a connection between mutations in *LRRK2* and visual perturbations^15–17^. A study in *Drosophila* showed that the G2019S mutation caused a significant change in contrast gain-control^18,19^. Furthermore, photoreceptor function has been shown to deteriorate in these flies due to excitotoxicity^16^. PD patients with LRRK2 mutations have been reported to perform worse in colour discrimination tasks than PD patients not carrying LRRK2 mutations^15,20^. In the same study, even nonsymptomatic carriers of the G2019S mutation showed a different response from controls in the Farnsworth-Munsell 100-hue task^15^.

In this study, visual evoked potentials (VEPs) were recorded in LRRK2-knockout rats to shed more light on the functional involvement of LRRK2 in visual processing. Evoked responses in the visual cortex and superior colliculus were recorded. VEP recordings were complemented by recordings of steady-state VEP (SSVEP). Visual stimulation was performed at different wavelengths to test for colour dependence of any changes, as suggested by the results of Marras and colleagues^15^. Furthermore, SSVEP recordings were used to assess changes in the oscillatory activity of the brain, such as the delta and alpha bands, which are often associated with the awake state. Alpha band oscillation is specific to the cortex, while delta oscillation occurs more broadly. Delta oscillation is hypothesized to have a thalamic origin^21^ and has been found to have increased power in a PD model where dopamine is depleted in the basal ganglia^22^.

The hypothesis of this study was that innate deficiency of LRRK2 would cause altered visual processing compared to that of wild-type rats. It was further hypothesized that the administration of the LRRK2 kinase inhibitor called PFE360^23^, would influence visual processing in wild-type rats but have no effect on visual processing in LRRK2 knockout rats.

## Methods

All animal experimentation was carried out in accordance with the European Communities Council Directive (86/609/EEC) and in accordance with Danish law on the care of laboratory animals. The protocols were approved by the Danish Animal Experiments Inspectorate (Forsøgsdyrstilsynet) prior to the initiation of the study under licence 2015-15-0201-00216. The study is reported in accordance with the ARRIVE guidelines.

### Animals

Fifteen LRRK2-KO female rats and 14 wild-type (WT) female rats weighing approx. 225 g (ten weeks of age) were obtained from SAGE laboratories, US. The LRRK2-knockout (KO) rat is a homozygous knock-out with a 10 bp deletion in exon 30, developed by SAGE laboratories in collaboration with the Michael J. Fox foundation. Rats were initially housed in pairs until surgery. After surgery, they were single-housed to prevent damage to the implants. They were housed in reverse daylight with a 12/12 light/dark cycle, lights off at 06:00. The home cages contained woodchip bedding and enrichment in the form of a red polycarbonate retreat and nesting material. Food enrichment in the form of various grains and dried plant parts, was provided once a week in the home cage. Food and water were available ad libitum*.* No animals were excluded from the study.

### Surgery

The animals were anaesthetized using subcutaneous injections of Hypnorm® (Lundbeck, Valby, Denmark), midazolam 5 mg/ml (B. Braun, Melsungen, Germany) and saline in a 2:1:1 ratio (2.0 ml/kg), yielding a final dose of 157 µg/kg fentanyl. Norodyl (carprofen 5 mg/kg) (ScanVet, Fredensborg, Denmark) and Noromox prolongatum (amoxicillintrihydrate 150 mg/kg) (ScanVet, Fredensborg, Denmark) were administered presurgery and for five days post-surgery. After induction of anaesthesia, the animals were mounted in a stereotactic frame, and Marcain (2.5 mg/ml bupivacaine, AstraZeneca, Albertslund, Denmark) was administered locally and subcutaneously prior to incision. A pulseoximeter (Nonin BV., NL) was attached to a hind paw to monitor heart rate and blood oxygenation. The coordinates were guided by the rat brain atlas by Paxinos and Watson^24^. Holes were drilled bilaterally for the visual cortex (VC) (AP: − 6, ML: ± 4) and superior colliculus (SC) (AP: − 6, ML: ± 1, DV: − 3.5) and unilaterally for the reference electrode (AP: + 8, ML: − 2) and the ground electrode (AP: − 2, ML: + 4). The electrodes for recording in the SC were E363/3/Spc stranded electrodes (PlasticsOne, VA, US). The other four electrodes were E363/20/2.4/S screw electrodes (PlasticsOne, VA, US). The electrode leads were collected in an MS363 plastic pedestal (PlasticsOne, VA, US) and fixed to the skull with RelyX™ Unicem dental cement (3 M, Copenhagen, Denmark) as a chronic implant. The skin was sutured around the implant. For 5 days after surgery, the animals were weighed daily and their welfare was checked by visual inspection of fur and activity level. The sutures were removed under sevoflurane anaesthesia after 5–7 days, depending on the healing of the animals. The animals were allowed two weeks of recovery before being subjected to any experimentation.

### EEG recording

The recordings were made with awake and active animals in a home cage with woodchip bedding. The housing cage was placed in a sound-attenuating cabinet shielded from light and electrical noise. During recordings, the animals were monitored via live video. The electrophysiological responses were recorded through a tether going via a brush commutator to a Brownlee amplifier model 410 (Brownlee Precision, CA, US) with the following settings: low-pass filter; 200 Hz, high-pass filter; 1 Hz, sampling rate; 1 kHz. The signal was recorded via a 1401 (CED, UK) using the software, Spike2 7.20 (CED, UK). Spike2 was used for recording as well as delivering stimuli.

The rats were exposed to 400 s of 1 Hz, 10 ms light flashes (lights ON) from LEDs placed 40 cm above the floor of the cage. The light intensity was 20 lx measured at the bottom of the cage. During the first recording, the rats were exposed to five different wavelengths of light: shortwave blue (SWB) (405 nm), blue (455–460 nm), green (525–530 nm), red (620–625 nm) and white 5050 SMD LEDs. The purpose was to investigate whether the knockout of *LRRK2* interacted with the wavelength of stimulation. The experimenter was accidentally unblinded to the genotype during the recording sessions as the VEP waveform from the LRRK2 deficient rats shows considerable oscillations relative to the wildtypes. Blue LEDs were chosen for further investigation, as this condition showed the largest cumulative difference between genotypes.

In addition to flash VEPs, steady-state VEPs (SSVEP) were recorded. The stimulus trains consisted of a 100 s train of 10 ms lights ON, presented at a rate of 14 Hz and followed the 400 s of 1 Hz flashes for each wavelength.

### Pharmacological LRRK2-kinase inhibition with PFE360

The rats received a single dose of PFE360 corresponding to 7.5 mg/kg, before administration PFE360 was dissolved in 10% captisol, and the pH was adjusted to > 2 using 1 M methanesulfonic acid. The solution was administered orally using a gavage in a randomized crossover study. Randomization was performed using the sample function in R (R 3.4.2) (RRID:SCR_000432). The concentration was based on previous research by Andersen and colleagues, showing that full kinase inhibition could be expected for 1–10 h after administration of this particular dose^13,14^. Similar to the study by Andersen and colleagues, the animals were not fasted before administration. All recordings were carried out 1 h after administration, as this is the brain C_max_ of PFE360^14^. Additionally, each stimulus session was repeated three times, run back-to-back to increase the statistical power of the study. The animals were prevented from sleeping during the recording session, by the experimenter tapping the sound-attenuating cabinet. To rule out time variance, the randomization of day one was repeated with a one-week interval. The final sequence became Day1–Day1–Day2, with the dashes representing a week. This made it possible to compare the amplitude and latency data of the first recording with those of the second, which showed that there was no significant difference under the vehicle condition in the LRRK2-KO group or the WT group (data not shown) indicating that time did not introduce any statistically significant changes.

A study on the functional consequences of washing out PFE360 was also conducted (shown in Supplementary Figs. S5–S8). Recordings were made in four animals at 1, 3, 6, 9 and 24 h after administration.

### Postmortem assessments

The animals were anaesthetized with 5% isoflurane and sacrificed by decapitation at 1 h (n = 8), 3 h (n = 8) and 6 h (n = 6) after administration of PFE360 on the last day of recording. Trunk blood and the cerebellum were sampled for exposure to confirm previously reported values for the T½ of PFE360^13^. The method for measuring the concentration of PFE360 are reported by Andersen and colleagues^14^. The cerebrum was used to histologically assess the location of the electrodes, data points would be excluded if the electrodes were not in the superior colliculus. No data points were excluded.

### Data analysis

The VEPs were computed by averaging across stimuli from − 0.2 to + 0.5 s relative to stimulation offset, using Spike2. This procedure averages out noise that is not time-locked to the stimulus. The naming of the peaks in the visual cortex was guided by Creel et al.^25^ and Meeren et al.^26^. The first positive deflection was named P1, the first negative deflection was named N1, the second positive deflection was named P2 and so on (peak designation is illustrated in Figure S1). For flash VEPs, the amplitude and latency of the peaks were extracted manually and analysed separately for each peak. Amplitudes were measured as voltage changes from baseline to maximum peak, and latency to peak was measured from flash offset to maximum peak.

The SSVEP has been suggested to provide a more robust response than the transient VEP, but the SSVEP does not provide temporal information, as it is a summation of previous responses. Consequently, it was analysed in the frequency domain^27^. An example waveform of the SSVEP is shown in Figure S2. The fast Fourier transform was computed using source code from Accusleep^28^ in MATLAB 2016a (Mathworks, MA, USA). The spectra were then averaged, so each 1-s epoch was treated as a repetition. Normalized power was computed as the power divided by the total power across the power frequency spectrum. The awake state is associated with bimodal activity in the delta band (0.5–4 Hz and the alpha band (8–12 Hz). Here, the alpha band is expected to be between 7 and 9 Hz, and for practical reasons, the delta band is defined in the range 1–2.6 Hz (see Fig. 2). The first harmonic of the stimulus occurs at the same frequency as the stimulus (14 Hz), and the second harmonic occurs at 28 Hz^27,29^. SSVEPs have been proposed to enable the distinction of cell types in the retina^18^ by examining the frequency bands of the signal; thus, the first harmonic should correspond to the response of the photo receptors, while the second harmonic should correspond to the response of the bipolar cells.

### Statistics

The extracted amplitude, latency and SNR data were analysed in R (R 3.4.2)^30^ via the Rstudio interface for R (Rstudio, MA, US).

Each animal had bilateral electrodes, doubling the number of data points in the statistical test. These double data points are not considered independent; therefore, the within-group correlation matrix is based on the individual animal, in the linear mixed model. The effects of colour or PFE360 on VEP and SSVEP peaks were evaluated by two-way repeated-measures ANOVA of the following model: data ~ genotype *(drug or colour) + error (ID). The backward variable elimination strategy developed by Hocking, was applied to prioritize the interactions and variables^31^. The analysis was carried out separately for each peak for each recording site, yielding 24 ANOVAs, and the results from these tests were adjusted with the false-discovery rate (FDR) method to correct for multiple comparisons. This was followed by Tukey’s post hoc test with FDR adjustment if *p* < 0.05.

## Results

### The difference between LRRK2-KO and WT depends on the wavelength of stimulation

**Figure 1:**
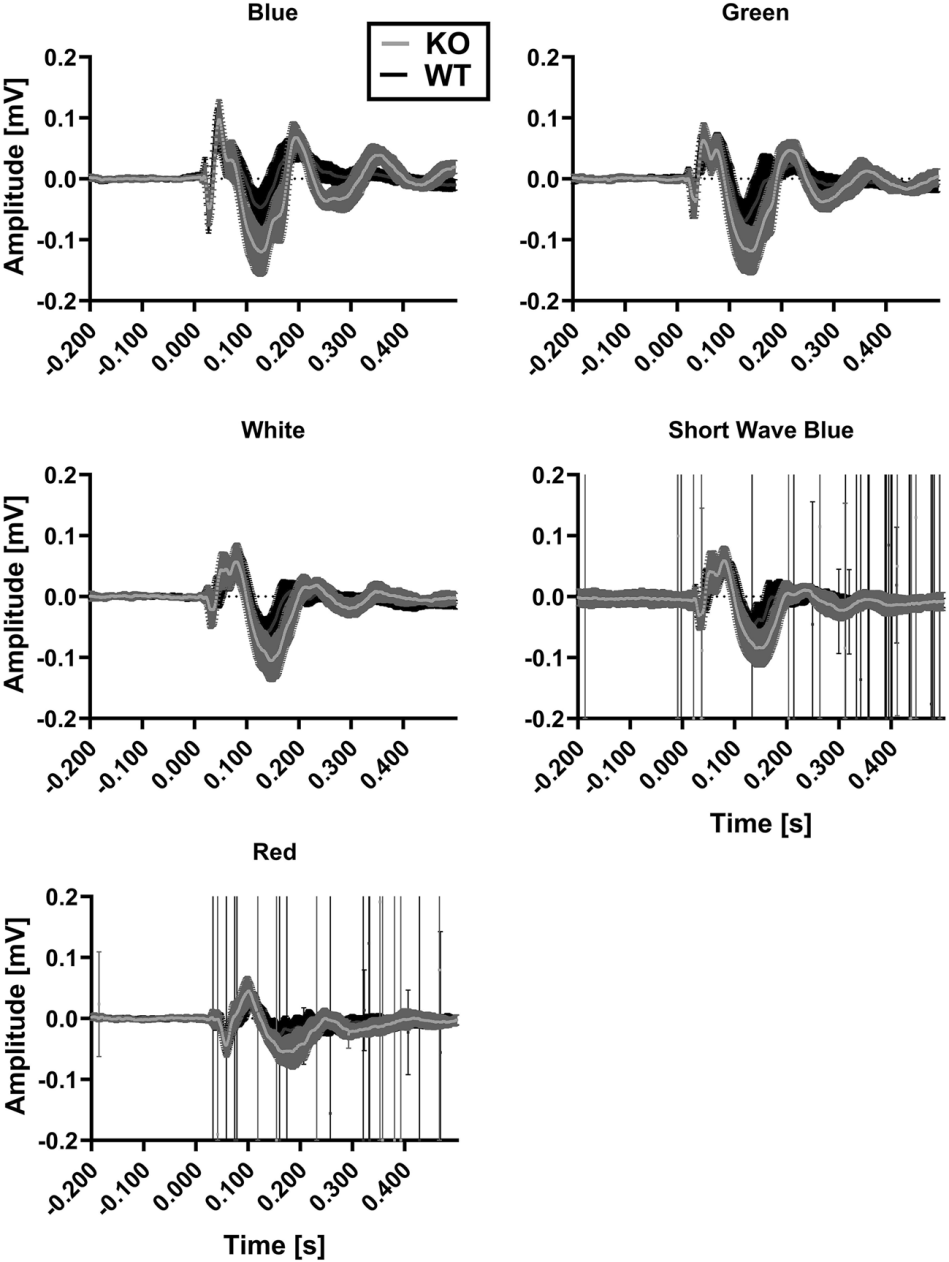
Grand averages of the visual cortex under five different wavelength conditions. The standard deviations are shown as shading. The grey line represents the response in the LRRK2 knock-out, and the black line represents that in the wild type. The difference between the KO group and the WT group varied with the wavelength of light stimulation. The difference between the means was largest for blue light.

LRRK2 has previously been connected to colour discrimination. Here, the animals were exposed to full-field light flashes at five different wavelengths, as shown in Fig. 1. The purpose of this part of the study was to find the wavelength most suitable for testing the effect of the LRRK2 inhibitor PFE360. Only the visual cortex (VC) data are shown here, while the superior colliculus (SC) data are shown in the supplementary material (Fig. S3). The responses were quantified for the green, blue and white conditions. The interaction between genotype and colour was significant for the peaks P1 (F(2,54) = 8.19, *p* value = 0.00280), N1 (F(2,54) = 5.96, *p* value = 0.0107), N2 (F(2,54) = 4.48, *p* value = 0.0277) and N3 (F(2,54) = 15.9, *p* value = 0.0007), suggesting that genotype differences are colour dependent. The difference between the KO and WT was quantified for the blue (cumulated mean difference was 0.143 mV), green (cumulated mean difference was 0.123 mV) and white (cumulated mean difference was 0.101 mV) conditions. In all cases the KO group showed the greater response. As the mean difference in the blue condition was the largest, the crossover with PFE360 was carried out using stimulation with this wavelength. The differences in the VEPs recorded in the SC were less pronounced.

### Changes in the wavelength of stimulation impact intrinsic oscillations in LRRK2-deficient rats

**Figure 2:**
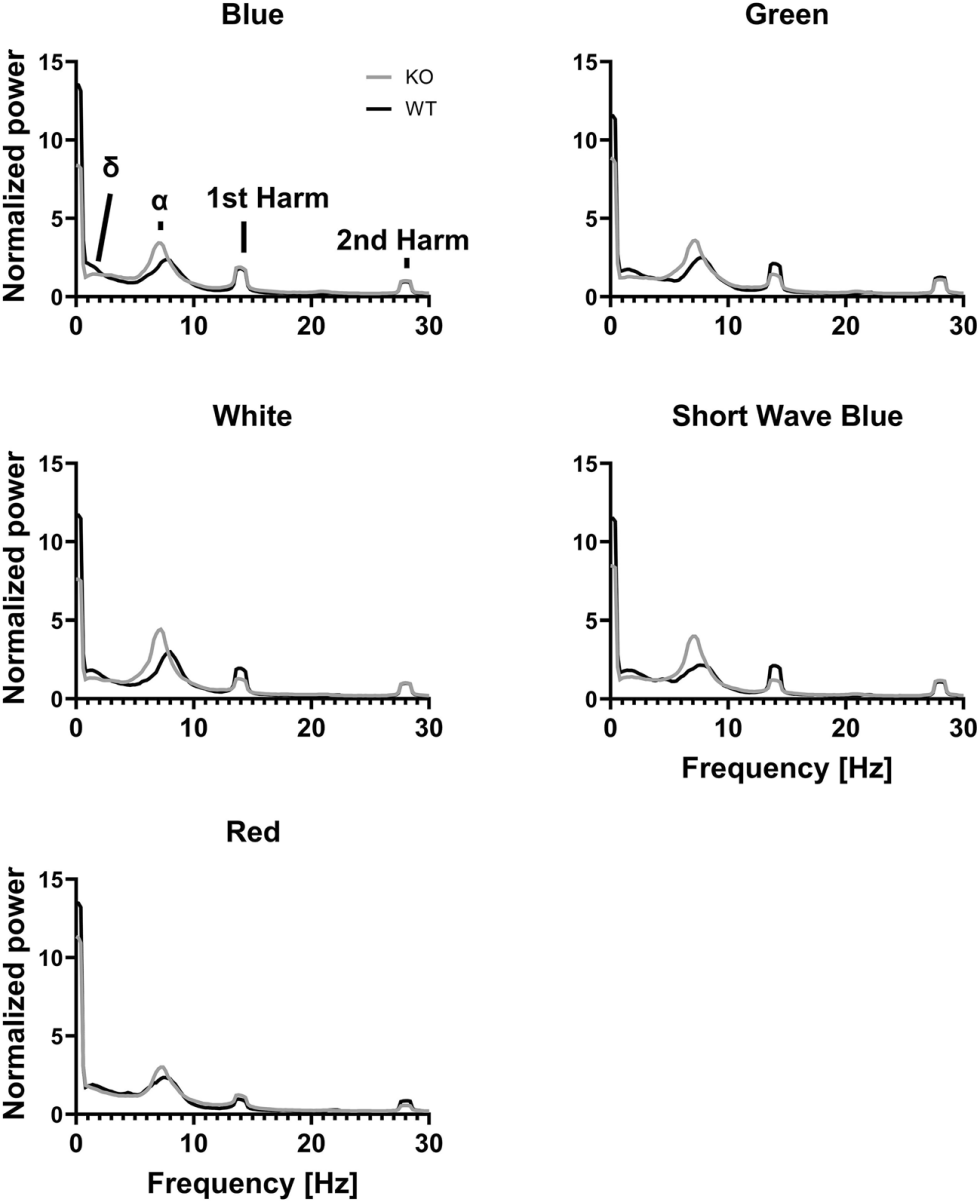
SSVEP is affected by the wavelength of stimulation. The power–frequency spectra are from SSVEP recordings from the visual cortex. The peaks are indicated in the top left panel. The intervals for determining the local maximum were defined as delta, 1–2.6 Hz; alpha, 4–11.6 Hz; 1st harmonic, 12–16 Hz; and 2nd harmonic, 26.6–29.8 Hz. Grey: KO, black: WT.

In addition to being useful in assessing visual responses, SSVEP recordings are also used to study intrinsic rhythms. The frequency spectra of the responses recorded in the visual cortex are shown in Fig. 2, while mean normalised power and standard deviation is shown in Table 1S. The responses recorded in the superior colliculus are shown and described in Fig. 4S and Table 2S.

The delta band showed similar results, with a significant interaction between colour and genotype F(4,108) = 0.01. The post hoc analysis revealed that the red condition for the KO group showed higher power compared to the other colour conditions. Additionally, the blue condition differed between the KO group and the WT group, with 1.64 ± 0.403, z = 4.07, *p* < 0.01.

The alpha band showed a significant interaction between colour and genotype F(4,108) = 3.39, *p* = 0.0117. The post hoc analysis showed that the LRRK2-KO group in the white condition had the higher power compared to the blue, green and red conditions. The red condition showed a lower power compared to the SWB condition.

For the 1st harmonic, there was a significant interaction between colour and genotype F(4,108) = 10.6, *p* < 0.0001. According to the post hoc test, there were differences within both the WT group, where red wavelength showed lower power compared to the other colours, and the LRRK2-KO group, where the blue condition showed higher power compared to red, SWB and white conditions. The two genotypes differed in the SWB condition with the KO group showing the lower power. For the 2nd harmonic, there was a significant effect of colour F(4,108) = 11.5, *p* < 0.0001. In the KO animals, the red condition showed lower power compared to the other conditions. For the WT group, the red condition caused lower power compared to the green condition. There were no significant effects on the peak frequencies. The statistical results are shown in Table 3S.

It is interesting that there is an effect of the colour condition on the intrinsic rhythm when the harmonics are the responses to flashing light. Noticeably, the effects occur primarily in the KO animals.

### Administration of the LRRK2 inhibitor PFE360 affects visual processing in both LRRK2-KO and WT rats

**Figure 3:**
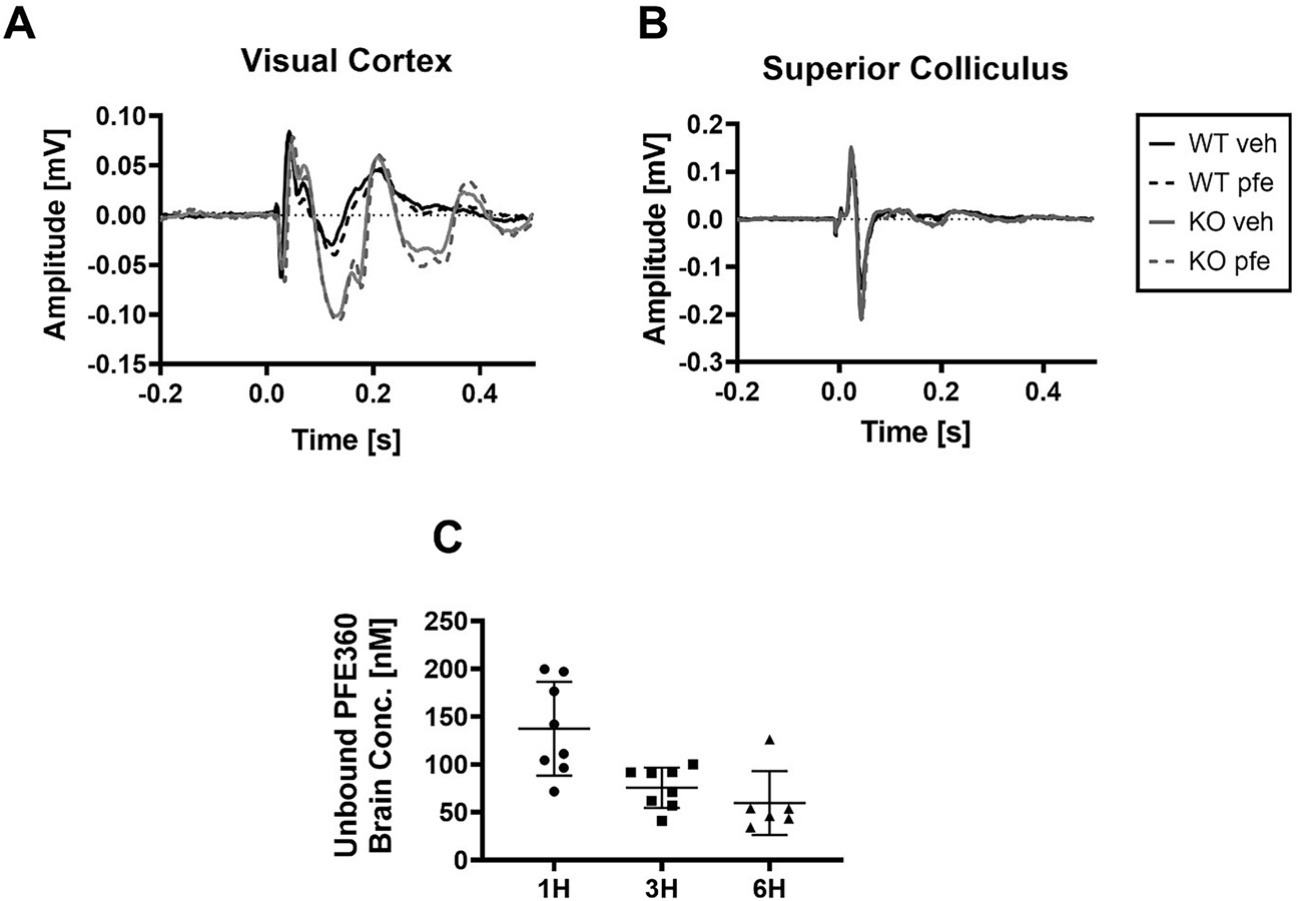
The effect of PFE360 on visual evoked potentials and pharmacological profile of PFE360. (**A**, **B**) show the four waveforms of WT rats one hour after administration of vehicle (black line) or PFE360 (dashed, black line) and LRRK2-KO rats after administration of vehicle (grey line) or PFE360 (dashed, grey line). The pretreatment time was 1 h. (**C**) shows the concentration of unbound PFE360 in the cerebellum at three time points after administration; here, both groups are pooled.

To study the effect of modulating LRRK2 activity, the LRRK2 kinase inhibitor PFE360 was administered to both LRRK2-KO and WT rats in a randomized crossover study. Figures 3A,B show the waveforms recorded from the visual cortex and the superior colliculus after administration of either vehicle or PFE360 and stimulation with blue light at a wavelength of 455–460 nm. Figure 3C shows the concentration of PFE360 at three time points after administration. The unbound concentration was 137.4 nM 1 h after administration, which is more than 10 times the IC_50_ of 2.3 nM reported by Andersen and colleagues^13^.

### The VEPs in the visual cortex were altered in LRRK2-KO rats following PFE360 administration

**Figure 4:**
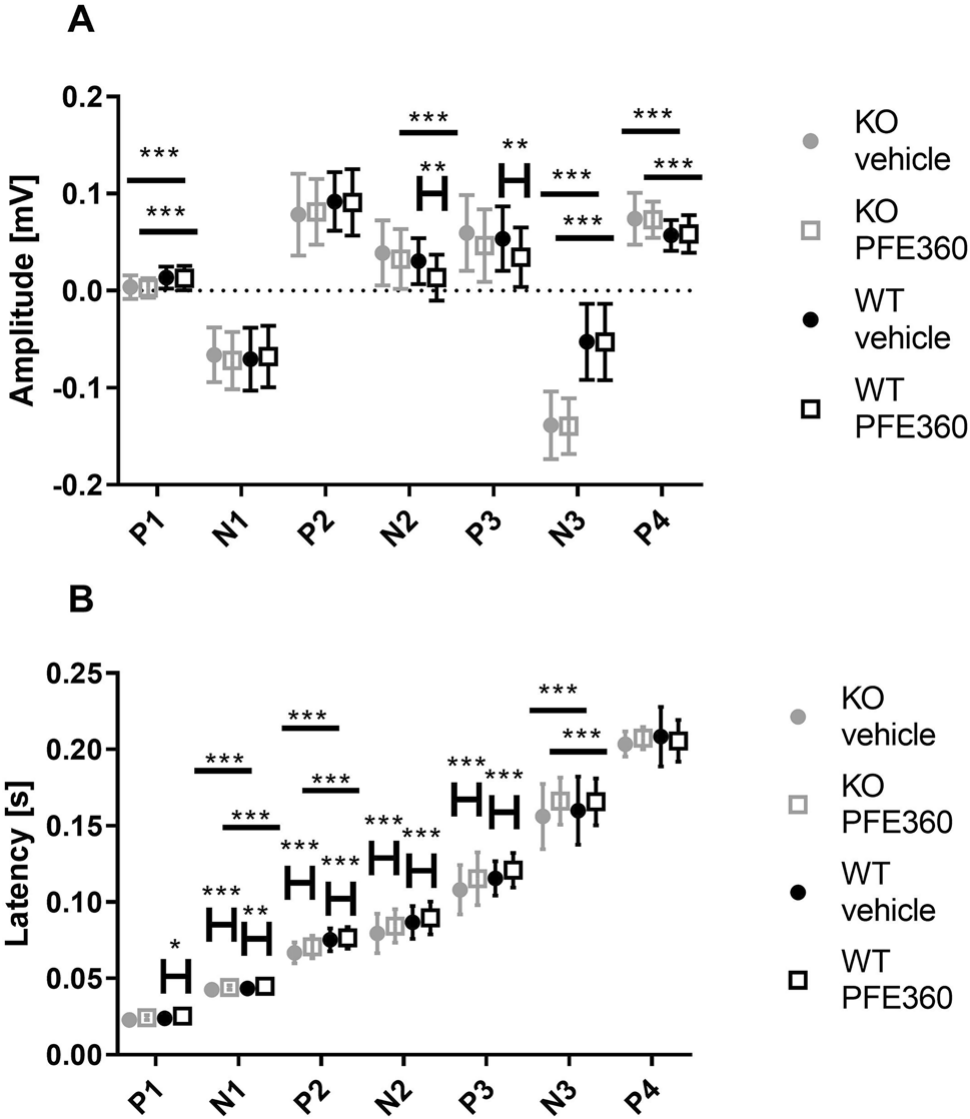
Amplitudes and latencies of the peaks in the waveform from the visual cortex shown as boxplots. (**A**) The amplitudes of all peaks are shown. Grey dot; vehicle condition KO, grey open box; PFE360 condition KO, black dot; vehicle condition WT, black open box; PFE360 condition WT. (**B**) Latency of the VEP peaks in the waveforms recorded from the visual cortex, shown as boxplots. Asterisks refer to results from the post hoc tests: *p < 0.05, **p < 0.01, ***p < 0.001.

The amplitudes of the VEPs obtained from the visual cortex are quantified in Fig. 4A, with the outcome of the Tukey post hoc test represented as asterisks. The results of the overall two-way ANOVAs are shown in Table 1. The amplitude of the peaks did not show any significant interaction between genotype and PFE360. For P1, N2, N3 and P4, there were significant effects of genotype, while for N2 and P3, there were effects of PFE360. The amplitude of the P1 peak was reduced by 0.0099 ± 0.0018 mV in LRRK2-KO rats compared to wild-type rats. The genotype effect on the amplitude of the N2 effect was not significant in the post hoc test. The N3 peak was significantly larger (0.086 ± 0.0055 mV) in LRRK2 KO rats than in WT rats. This corresponded to the amplitude on N3 in the LRRK2-KO group being 2.6 times the amplitude of N3 in the wild-type group. The amplitude of the P4 peak was significantly increased by 0.015 ± 0.0032 mV in LRRK2-KO rats compared to wild-type rats. PFE360 administration reduced the amplitudes of the N2 and P2 peaks in WT rats, with − 0.017 ± 0.0045 mV for N2 and − 0.019 ± 0.0056 mV for P3, while there was no detectable effect of the drug on the VEP peaks in the LRRK2-KO rats.

**Table 1:** The overall statistical results from ANOVA evaluating differences in the amplitude and latencies of the visual cortex waveforms as resulting from the variables gene (WT and LRRK2-KO rats) and drug (vehicle and PFE360).

		Two-way ANOVA		
			F value	Adjusted *p* value (if *p* < 0.05)
Amplitude of peaks in the visual cortex				
P1	Gene	F(1,27)	13	0.0014
	Drug	F(1,304)	0.69	
N1	Gene	F(1,27)	0.033	
	Drug	F(1,304)	0.88	
P2	Gene	F(1,27)	0.65	
	Drug	F(1,304)	0.036	
N2	Gene	F(1,27)	4.3	0.049
	Drug	F(1,304)	23	0.00021
P3	Gene	F(1,27)	1.1	
	Drug	F(1,304)	33	0.00021
N3	Gene	F(1,27)	64	0.00021
	Drug	F(1,304)	0.45	
P4	Gene	F(1,27)	9.9	0.006
	Drug	F(1,304)	0.0079	
Latency of peaks in the visual cortex				
P1	Gene	F(1,27)	0.079	
	Drug	F(1,304)	16	0.00021
N1	Gene	F(1,27)	16	0.00064
	Drug	F(1,304)	117	0.00021
	interaction	F(1,304)	15	0.00036
P2	Gene	F(1,27)	53	0.00021
	Drug	F(1,304)	54	0.00021
N2	Gene	F(1,27)	0.80	
	Drug	F(1,304)	96	0.00021
P3	Gene	F(1,27)	0.010	
	Drug	F(1,304)	69	0.00021
N3	Gene	F(1,27)	15	0.0008
	Drug	F(1,304)	20	0.00021
P4	Gene	F(1,27)	0.77	
	Drug	F(1,304)	10	

The *p* value is displayed after FDR adjustment.

Figure 4 shows the grand means of the VEP latencies from the visual cortex waveforms. The results of two-way ANOVAs are shown in Table 1. Here, the interaction between genotype and drug on the latency of the N1 peak, was statistically significant. Peaks P2 and N3 showed significant effects of genotype. Regarding the effect of genotype, the latencies of N1 and P2 in the LRRK2-KO were increased by 2.5 ms and 5 ms, respectively, and that of the N3 peak was increased by 20 ms relative to the latencies in the WT. Peaks P1, P2, N2, P3 and N3 showed significant effects of PFE360. PFE360 caused increases in latencies ranging from 1.2 to 5.6 ms in both WT and LRRK2-KO rats. No statistically significant changes were observed in the P4 peak.

Since the LRRK2-KO group does not express LRRK2, it can be assumed that the effects in these animals are caused by off-target effects of PFE360. Consequently, the only effects that may be related to LRRK2 are the changes in the amplitude of N2 and P3, along with those in the latency of P1.

### PFE360 induced changes in the VEPs recorded from the superior colliculus

**Figure 5:**
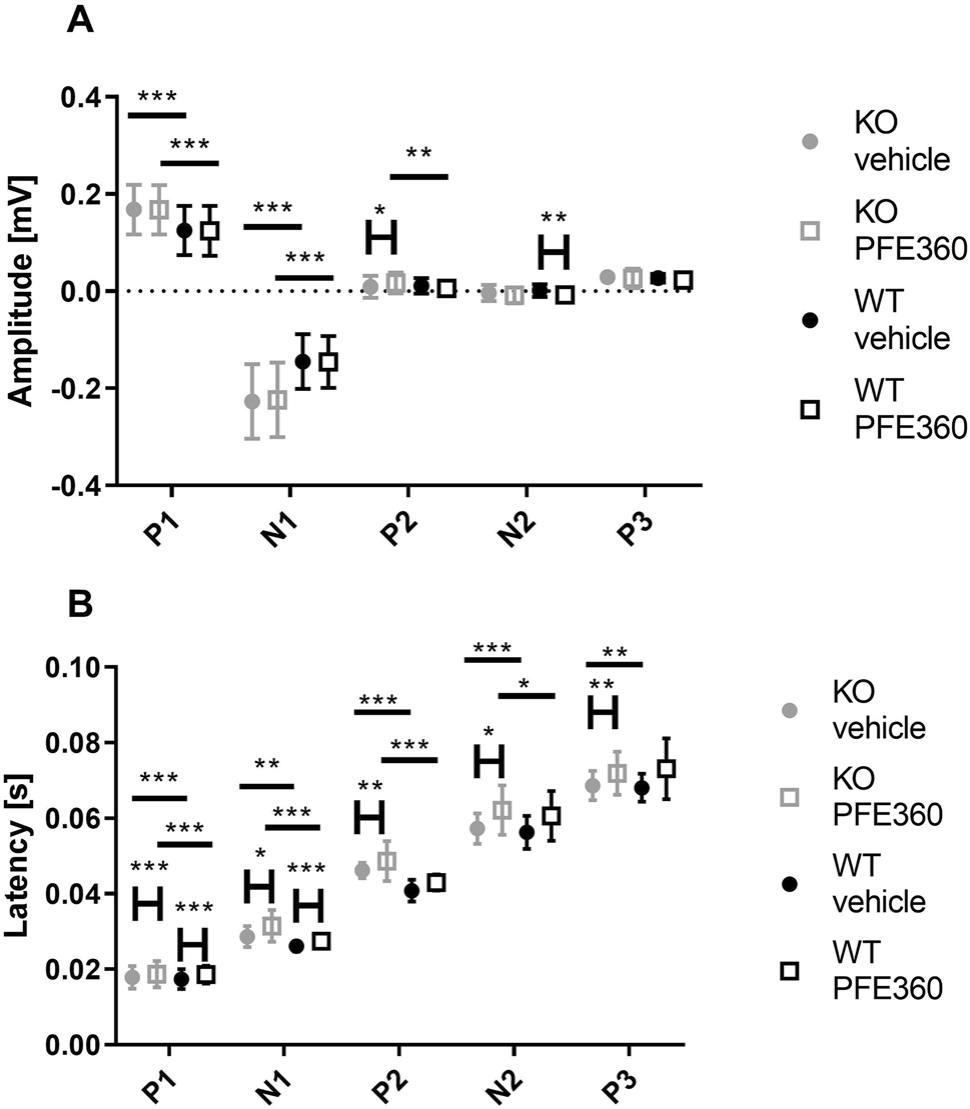
Amplitudes and latencies of visual evoked potentials measured in the superior colliculus. (**A**) Amplitudes of each peak displayed as boxplots. (**B**) Latencies of visual evoked potentials in the superior colliculus, shown as boxplots. Asterisks refer to results from the post hoc tests: *p < 0.05, **p < 0.01, ***p < 0.001.

The grand average amplitudes of the peaks recorded from the superior colliculus are displayed in Fig. 5A, with the outcome of the post hoc test indicated by asterisks. The results of the overall two-way ANOVA for the effect of genotype and drug are shown in Table 2. The amplitude of P2 showed a significant effect of the interaction between genotype and PFE360: an increase in amplitude was caused by the drug in the LRRK2-KO group (0.0083 ± 0.0030 mV) but not in the WT. Both P1 and N1 showed significant genotype effects; P1 in LRRK2-KO rats was increased by 0.043 ± 0.0083 mV, while N1 in LRRK2-KO rats showed a negative increase in amplitude by 0.08 ± 0.011 mV. PFE360 significantly changed the amplitudes of P2 in the LRRK2-KO rats and N2 in the WT rats. N2 showed a significant decrease in amplitude in WT rats (0.0055 ± 0.0032 mV) following PFE360 treatment. This difference may be the only one resulting from acute inhibition of LRRK2. The post hoc test did not show changes in the amplitude of P3.

**Table 2:** Results from two-way ANOVA testing the variables gene (WT and LRRK2-KO rats) and drug (vehicle and PFE360).

		Two-way ANOVA		
			F value	Adjusted *p* value (if *p* < 0.05)
Amplitude of peaks in the superior colliculus				
P1	Gene	F(1,27)	6.5	0.019
	Drug	F(1,273)	0.0070	
N1	Gene	F(1,27)	14	0.0012
	Drug	F(1,273)	0.065	
P2	Gene	F(1,27)	1.7	
	Drug	F(1,273)	0.87	
	interaction	F(1,273)	13	0.00064
N2	Gene	F(1,27)	0.96	
	Drug	F(1,273)	20	0.00021
P3	Gene	F(1,27)	0.66	
	Drug	F(1,273)	5.5	0.021
Latency of peaks in the superior colliculus				
P1	Gene	F(1,27)	8.3	0.009
	Drug	F(1,273)	137	0.00021
N1	Gene	F(1,27)	3.2	
	Drug	F(1,273)	84	0.00021
P2	Gene	F(1,27)	19	0.00036
	Drug	F(1,273)	14	0.00036
	interaction	F(1,273)	3.9	0.049
N2	Gene	F(1,27)	7.3	0.013
	Drug	F(1,273)	12	0.00076
P3	Gene	F(1,27)	3.1	
	Drug	F(1,273)	25	0.00021

Effects on the amplitudes and latencies of visual evoked potentials from the superior colliculus were evaluated. The p values were adjusted using the FDR for all 24 ANOVAs.

The grand average latencies of the superior colliculus are shown in Fig. 5B, with brackets and asterisks indicating the outcome of the post hoc test. The results of the overall two-way ANOVAs are shown in Table 2. The latency of P2 showed an effect of the interaction of genotype and PFE360. There were significant effects of genotype on P1 and N2. The post hoc test revealed effects of genotype on N1. For both P1 and N1, these effects were in the range of 1–3 ms. P2 and N2 showed an effect of genotype, and decreases in latencies ranging from 5 to 9 ms were observed in LRRK2-KO rats. There was an effect of PFE360 on all peaks. PFE360 increased the latency of P1 and N1 in both WT and LRRK2-KO rats by approximately 2 ms and increased the latency of the later peaks of the VEP; the latencies of P2 and N2 in LRRK2-KO rats were increased by 3–6 ms. For P3, there was an effect of genotype, with the KO rats being 7.4 ± 2.3 ms faster than the WT rats in the vehicle condition. The addition of the drug normalized this effect in the LRRK2-KO rats by increasing the latency by 7.4 ± 2.3 ms.

### LRRK2 modulation affects intrinsic oscillations rather than SSVEPs

**Figure 6:**
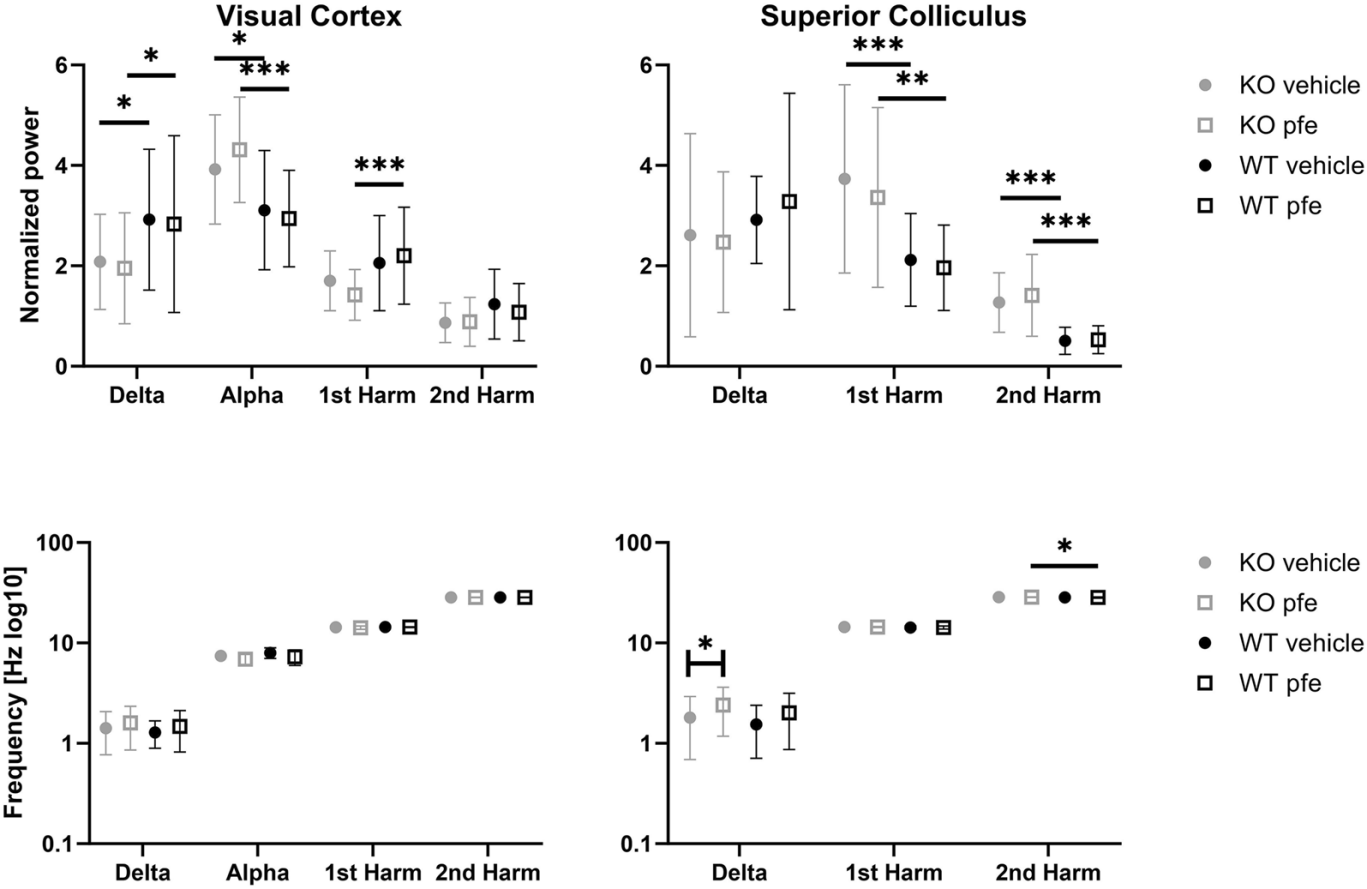
Normalized power and peak frequencies of SSVEPs recorded from the visual cortex and superior colliculus shown as boxplots. There was a trend toward an interaction between gene and PFE360 for the first harmonic of the visual cortex. There was a statistically significant effect of gene on the second harmonic of the superior colliculus. Asterisks refer to results from the post hoc tests: *p < 0.05, **p < 0.01, ***p < 0.001.

In addition to the VEPs, the SSVEPs were measured in the visual cortex and superior colliculus. The normalized power and peak frequencies of the intrinsic rhythms and harmonics are displayed in Fig. 6. Descriptive statistics can be found in Table 4S. Two-way ANOVA testing for the effects of genotype and PFE360 was carried out for each of the peaks of interest, and the results are displayed in Table 3. For cortical SSVEPs, two-way ANOVA of the normalized power showed significant genotype effects on the delta band, the alpha band and the first harmonic. The power in the delta band was decreased in the LRRK2-KO group (vehicle 0.838 ± 0.285, PFE360 0.883 ± 0.285). In the alpha band, the power in the LRRK2-KO group was increased (vehicle 0.813 ± 0.271 PFE360 1.37 ± 0.271). In the 1st harmonic, the power in the PFE360 condition was decreased by 0.779 ± 0.185 relative to the WT. There were no significant changes in the peak frequency. In the superior colliculus, there was genotype effects on the 1st and 2nd harmonic specifically an increase in power in the LRRK2-KO rats compared to the WT rats. The power of the 1st harmonic was increased for the LRRK2-KO group (vehicle 1.61 ± 0.427 PFE360 1.4 ± 0.427), and similar effects were observed for the 2nd harmonic (vehicle 0.765 ± 0.156, PFE360 0.883 ± 0.156). PFE360 increased the peak frequency of the delta band for the KO group by 0.596 ± 0.223, and a genotype effect on the 2nd harmonic was observed: the LRRK2-KO group in PFE360 condition showing a decrease of 0.19 ± 0.0648.

**Table 3:** F values from two-way ANOVA of model power = PFE360*genotype + (error(ID)).

F values of 2-way ANOVA			Drug F(1,143)	Genotype F(1,27)
Visual cortex	Normalized power	Delta		F = 18.2, *p* = 0.0008
		Alpha		F = 23.0, *p* = 0.0005
		1. Harm		F = 14.7, *p* = 0.0018
		2. Harm		F = 4.46, *p* = 0.0471
	Peak frequency	Delta		
		Alpha	F = 19.7, *p* = 0.0005	F = 6.9, *p* = 0.021
		1. Harm		F = 4.48, *p* = 0.0471
		2. Harm		F = 4.48, *p* = 0.0471
Superior colliculus	Normalized power	Delta		
		1. Harm		F = 14.8, *p* = 0.0018
		2. Harm		F = 33.9, *p* = 0.0005
	Peak frequency	Delta	F = 10.9, *p*= 0.0026	
		1. Harm		
		2. Harm	F = 4.0, *p* = 0.0471	F = 6.5, *p* = 0.0229

*P* values were adjusted with the FDR. PFE360 affects the peak frequency of intrinsic rhythms.

In general, knocking out LRRK2 caused opposite effects on VEP levels in the visual cortex and the superior colliculus, with longer latencies in the visual cortex but shorter latencies in the superior colliculus. In the visual cortex, the amplitude of P1 was lower, while the amplitudes of N3 and P4 were higher; conversely, in the superior colliculus, the amplitudes of P1 and N1 were increased, and there was no effect on the later peaks.

Administration of PFE360 caused longer latencies, while the effects on amplitude were more ambiguous in the visual cortex—PFE360 caused smaller amplitude effects for N2 and P3. In the superior colliculus, the amplitudes of P2 and N2 were larger. The LRRK2 inhibitor did not cause any significant changes in the power or frequency of the SSVEPs.

## Discussion

The purpose of the study was to investigate the role of LRRK2 in visual processing. VEPs and SSVEPs were recorded from the superior colliculus and the visual cortex of both LRRK2-KO and wild-type rats. Subsequently, the LRRK2 kinase inhibitor PFE360 was administered in a randomized crossover design. Previously, the knockout model has been used in toxicological studies of the use of LRRK2 inhibition as a therapy for PD. These studies have primarily shown adverse effects on the lung and kidneys^11,32^; here, we report changes in the electrophysiology of the visual system both VEPs and SSVEPs along with changes in intrinsic oscillations.

There was a significant difference in the overall VEP waveform between the LRRK2 knockout and wild-type rats. The magnitude of this difference depended on colour. Although different species have different pigments in the cones of the retina, colour perception differences related to LRRK2 seem to apply to rodents as well as humans and flies^15,16^. Curiously, the difference is largest when blue light is used. Rats primarily have cones with pigment sensitive to light in the green part of the spectrum at 500–520 nm^33^, and approximately 7% of those cones also express a pigment sensitive to the ultraviolet part of the spectrum at 370 nm^33^. In this study, the blue LED had a wavelength in the range of 455–460 nm, and the green LED had a wavelength in the range of 525–530 nm, demonstrating that the largest difference was not found at the peak sensitivity of rat cones. Interestingly, human patients with LRRK2-linked PD perform worse in the Farnsworth-Munsell 100-hue test than patients with idiopathic PD. The same effect was detected when comparing G2019S carriers with non-mutation carriers from the same families^15^. Unfortunately, there has been no EEG study on these patients studying whether the amplitude of the VEP correlates with the ease of discrimination. The results from this study show that the difference between the presence and absence of LRRK2 is the largest in the blue part of the spectrum.

Another interesting point emerges when looking at the SSVEP results: the colour conditions impact the LRRK2-KO group more than the WT group, indicating that the responses differ more in the KO groups. In particular, detectable differences in the delta and alpha oscillations occur in the LRRK2-KO group and not in the WT group. Taken together with the human data, these data show that LRRK2 knockout causes higher sensitivity to wavelength, while a gain-of-function mutation causes less sensitivity to wavelength. This could potentially mean that colour discrimination can be used as a biomarker for the function of LRRK2^20^. A study by Galter and colleagues showed that LRRK2 colocalized with dopaminoceptive regions of the brain^1^. One could speculate that this may be the same for the retina, where LRRK2 has also been detected^34^. However, dopaminergic cells in the retina have primarily been shown to form synapses with cells belonging to the rod pathway^35^. The rod pathway transmits luminance information, which corresponds poorly to the wavelength dependence shown in this study. Glutamate seems a far more likely candidate, as it is important in the cone pathway^36^. Other studies have shown an interaction between LRRK2 and the glutamatergic system^9,14,37^. These studies focused on overexcitation and excitotoxicity related to the G2019S GOF mutation^38^. The LRRK2 kinase aids in vesicle transport via interactions with Rab^9^; without LRRK2, this process may be less well regulated.

To further study the role of LRRK2 in the integrity of the visual system, blue light VEP and SSVEPs were recorded from the VC and SC. Overall, LRRK2 deficiency gave rise to larger amplitudes in the late peaks of the VEP from the VC and early peaks of the VEP from the SC. The latency measured in the VC is shorter in the LRRK2-KO group than in the WT group, while that in the SC is longer in the LRRK2-KO group in the same comparison. Hence, the transmission of visual information is affected differentially at the two recording sites when LRRK2 is knocked out. In the adult rodent brain, LRRK2 is enriched in the subthalamic nucleus and the VC but not in the SC^39–41^. This expression pattern could potentially explain why the differences between WT and LRRK2-KO in the overall VEP were more pronounced in the VC, indicating that differences in the colliculi are the result of a developmental deficiency of LRRK2. Furthermore, there were consistent and robust peaks detected after P4 in the VEP of the VC in the KO animals, suggesting that the absence of LRRK2 affects clearance from the synaptic cleft^9^. These extra oscillations did not appear in the wild-type group after administration of PFE360, suggesting that the oscillations were not caused by an acute lack of LRRK2 kinase activity. Additionally, the responses in the SC have higher amplitudes, indicating synchronization of more postsynaptic potentials; however, longer latencies indicate less efficient synchronization. Synchrony is an interaction between excitation and inhibition^21^. Inhibition can increase synchrony because a period of inhibition increases the probability of firing^42^, which might be the case here, as the system does not appear more excitable. Inhibition in turn can also be a side effect of slower glutamate release, which again may impact the oscillatory profile, e.g., alpha oscillations in the LGN of cats^43^, depending on the strength of mGluR1 activation. In the VC, there was an increase in alpha band power, with a decrease in delta band power, but decreased the power of the 1st harmonic. Additionally, the alpha band seems to be shifted to a slower frequency; however, this is not statistically significant. In the SC, the power of the two harmonics was increased.

LRRK2 is expressed in neural progenitor cells, suggesting a role in neurodevelopment. An in vitro study of LRRK2 deficiency in mouse embryonic stem cells reported changes in neurite outgrowth, possibly via retinoic acid interaction^44^. Furthermore, an in vivo study detected LRRK2 in the ventricular and subventricular zones associated with neurogenesis in tissue from murine embryos^2^, and changes in dendrites have been observed in a kinase knock-in model of LRRK2^10^. Collectively, these results suggest a role for LRRK2 in the development of neurons. These studies were carried out in mice, but LRRK2 is well conserved between mice and rats^45^.

The LRRK2 kinase inhibitor PFE360 affected the electrophysiological responses in both LRRK2-KO rats and WT rats. This inhibitor was described by Baptista and colleagues and has been reported to have minor off-target effects^23^. The dose applied in the present study has been shown to specifically target the kinase domain of LRRK2^13^. An acute dose of PFE360 caused longer latencies in both the VC and the SC of the KO group, similar to the effect in the wild-type rats, which suggests off-target effects of the LRRK2 kinase inhibitor, as reported by Thirstrup and colleagues^12^. However, LRRK2 dependency can be inferred for changes occurring only in wild-type rats, e.g., decreases in the amplitude of N2 and P3 in the VC. Additionally, PFE360 had a negative effect on the amplitude N2 of SC in the VEP. The decrease in amplitudes suggests a lower degree of synchronization of postsynaptic potentials.

Testing the vehicle condition twice in the same animals showed that the week between the first test and the crossover did not induce changes in the waveform of the VEP, demonstrating robustness in the measurement of the VEP. Adding to this robustness, the wash-out pilot with PFE360 showed that the induced changes were statistically significant with just four animals. This strengthens the conclusion that the changes in the VEP observed in the knockout rats are induced by PFE360. As the drug primarily affects latency of the responses, the absence of an effect on the SSVEP is not surprising, as the SSVEP is analysed in the frequency domain and therefore not directly impacted by changes in latency. Additionally, the free fraction one hour after administration was 50 times higher than the IC_50_; therefore, the probability of off-target effects was quite high. Some of the possible off-target kinases are MST 1/2, RSK2^23^, MAP3K5, MAP3K15 and MAP3K6^12^. These enzymes have very diverse functions, but MAP3K5 has been associated with stress-induced death of retinal ganglion cells in glaucoma^46^. Whether acute inhibition of MAP3K5 can affect visual processing remains to be studied.

A limitation of this study is that the function and expression of LRRK2 varies over time^2,41^, and this study only included one timepoint in adulthood. There are several indications that LRRK2 may be more important earlier in life; therefore, further research into the effects of LRRK2 on changes in developmental trajectory is still needed. Another potential limitation is the study only including one sex, though LRRK2-mutations have not indicated any interaction with sex it still requires more research to study if there is an interaction. In addition, more focus on LRRK2 and glutamate may also improve the understanding of the function of LRRK2.

In conclusion, this study demonstrated that LRRK2 is important for the development of signal transmission in the visual system and that it might play different roles in cortical regions and subcortical structures. In addition, the magnitude of the changes in the VEPs depended on the wavelength of stimulation, and alterations in the LRRK2-KO group were not induced in the WT group by acute administration of an LRRK2 kinase inhibitor. Further investigation of the underlying mechanism and the time dependence of these differences may help elucidate the consequences of LRRK2 modulation.

### Supplementary Information

Supplementary Information.

## Data Availability

Preprocessed data are available on GIN: 10.12751/g-node.4vv9rl.
