# Supplementary material for: Knocking out the LRRK2 gene increases sensitivity to wavelength information in rats
Source: Sci Rep. 2024 Feb 29;14:4984. doi: 10.1038/s41598-024-55350-9 (PMC10904730; doi:10.1038/s41598-024-55350-9)
Supplement: Supplementary file 1 — Supplementary Information. [file 41598_2024_55350_MOESM1_ESM.pdf]

Supplementary data for the manuscript: Knocking out the LRRK2 gene increases sensitivity to wavelength information in the rat

Author: Freja Gam Østergaard

Designation of peaks in VEP trace

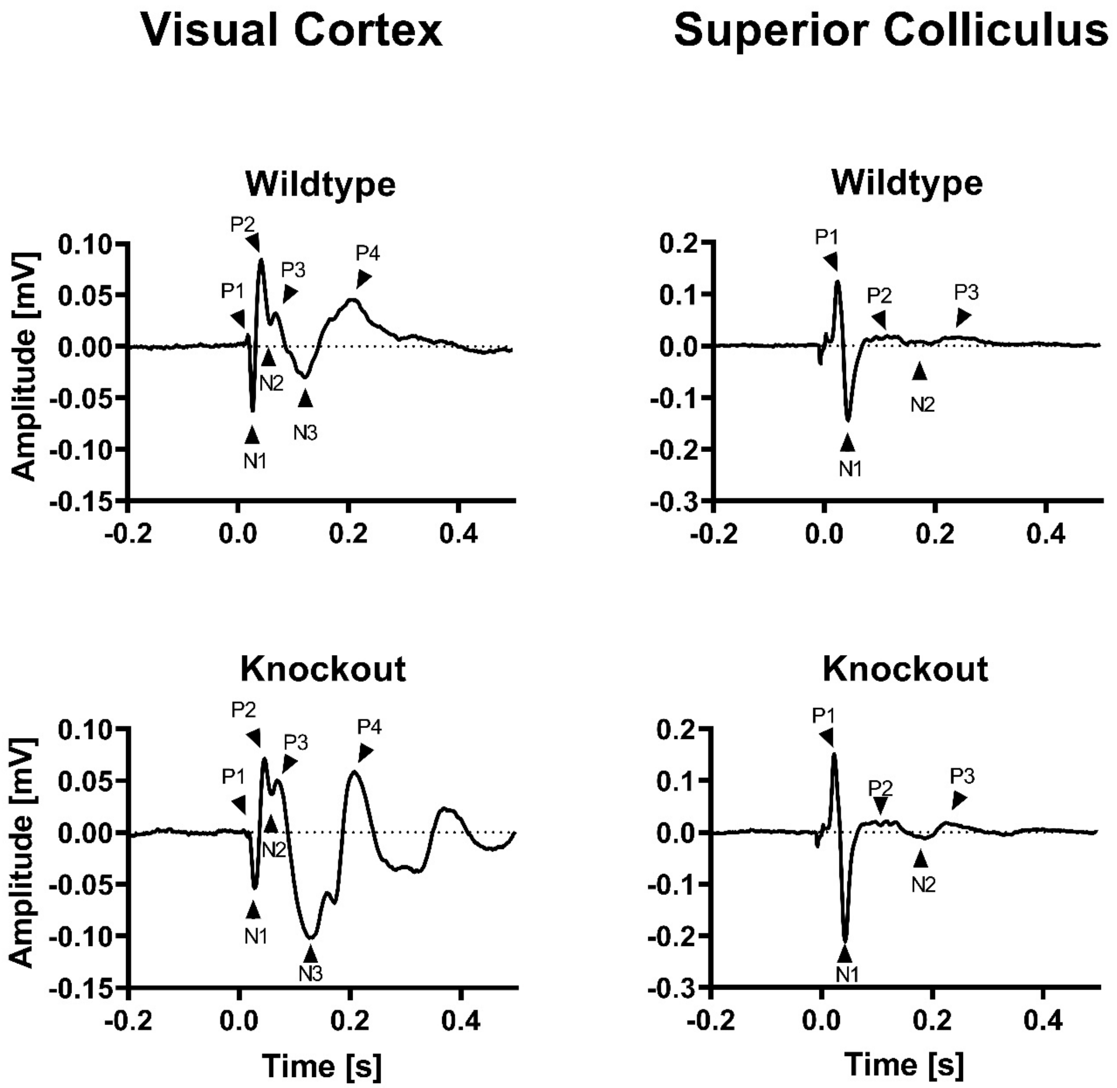

Figure S 1 Example peak designation of VEP traces measured from the visual cortex and superior colliculus in wildtype and KO rats.

Example of SSVEP response

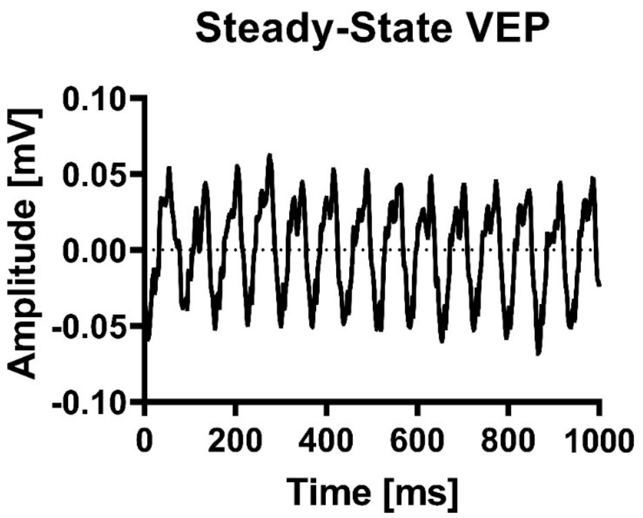

Figure S 2 Example of SSVEP recorded from the superior colliculus.

Pilot of interaction between genotype and wavelength condition

The purpose of this pilot was to test for interactions between wavelength of the stimulus presented and the genotype of the animal. This was investigated by recording the VEP of both wildtypes and LRRK2-KO during five different wavelength conditions:

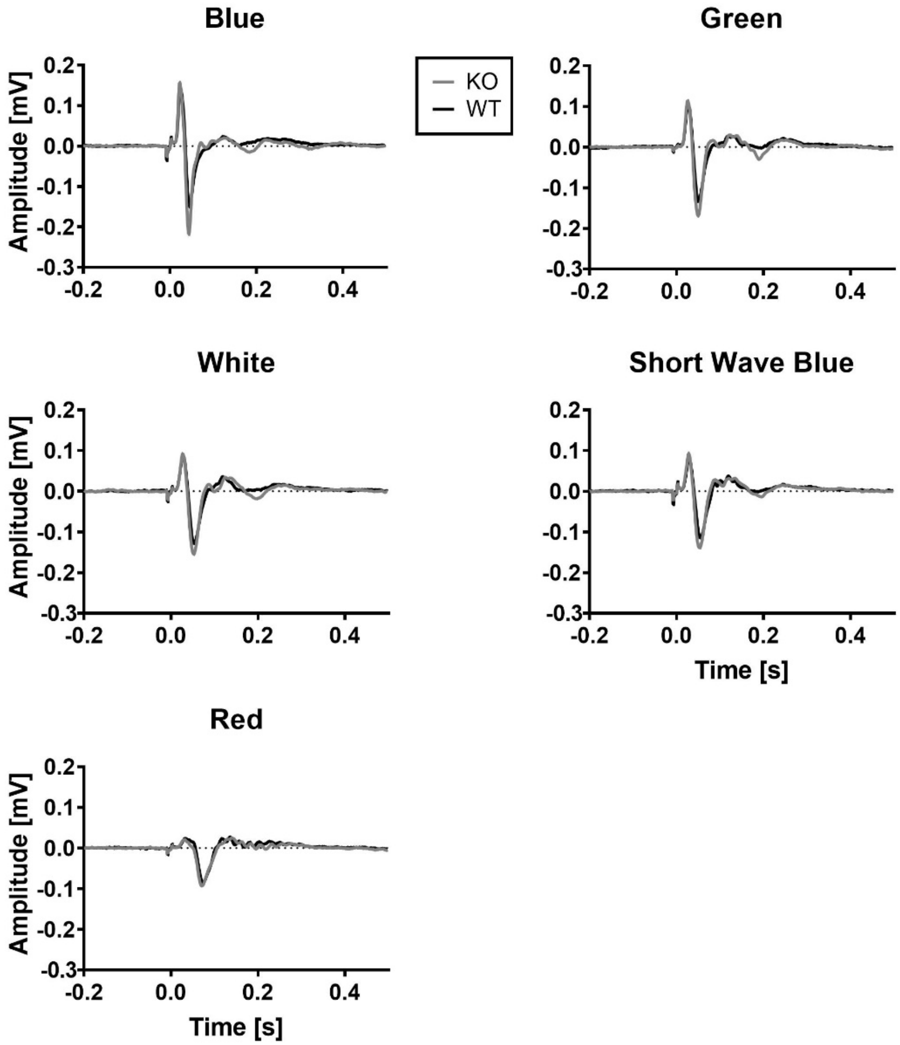

Figure S 3 Grand average waveforms from the superior colliculus recorded during five different wavelength conditions. The grey is the LRRK2 knock-out and black is the wildtype.

Table 1S SSVEP Visual cortex. Normalized power and frequency as mean (standard deviation). SWB: short-wave blue, WT: wildtype, KO: knock-out.

Visual Cortex		WT	N=14	KO	N=15
Peak	Colour	power	frequency	power	frequency
Delta	SWB	2.886 (0.625)	1.243 (0.485)	2.139 (0.528)	1.36 (0.551)
	Blue	3.832 (2.287)	1.129 (0.3)	2.189 (0.566)	1.507 (0.709)
	Green	2.788 (0.796)	1.171 (0.35)	2.21 (0.804)	1.2 (0.414)
	White	2.965 (0.724)	1.086 (0.232)	2.002 (0.748)	1.28 (0.549)
	Red	3.228 (0.827)	1.171 (0.414)	3.272 (1.617)	1.267 (0.494)
Alpha	SWB	2.872 (1.221)	7.143 (1.589)	4.318 (1.452)	7.453 (0.597)
	Blue	2.837 (0.972)	8.043 (0.827)	3.579 (1.086)	7.52 (0.361)
	Green	3.078 (1.195)	7.814 (1.157)	3.909 (1.191)	7.52 (0.439)
	White	3.562 (1.334)	8.257 (0.644)	4.863 (1.796)	7.52 (0.42)
	Red	2.946 (0.905)	7.657 (0.936)	3.173 (1.193)	7.493 (0.985)
1. Harmonic	SWB	2.126 (0.73)	14.257 (0.094)	1.225 (0.558)	14.147 (0.563)
	Blue	1.8 (0.558)	14.343 (0.094)	1.901 (0.676)	14.28 (0.126)
	Green	2.125 (0.807)	14.271 (0.127)	1.466 (0.47)	14.107 (0.544)
	White	1.962 (0.654)	14.314 (0.103)	1.37 (0.464)	14.107 (0.544)
	Red	0.973 (0.229)	14.229 (0.133)	1.253 (0.431)	13.973 (0.618)
2. Harmonic	SWB	1.101 (0.425)	28.443 (0.16)	1.179 (0.495)	28.413 (0.141)
	Blue	0.96 (0.455)	28.371 (0.107)	1.02 (0.431)	28.373 (0.07)
	Green	1.24 (0.61)	28.371 (0.107)	1.099 (0.408)	28.427 (0.103)
	White	0.989 (0.348)	28.471 (0.099)	1.014 (0.515)	28.413 (0.092)
	Red	0.861 (0.384)	28.414 (0.146)	0.567 (0.196)	28.4 (0.151)

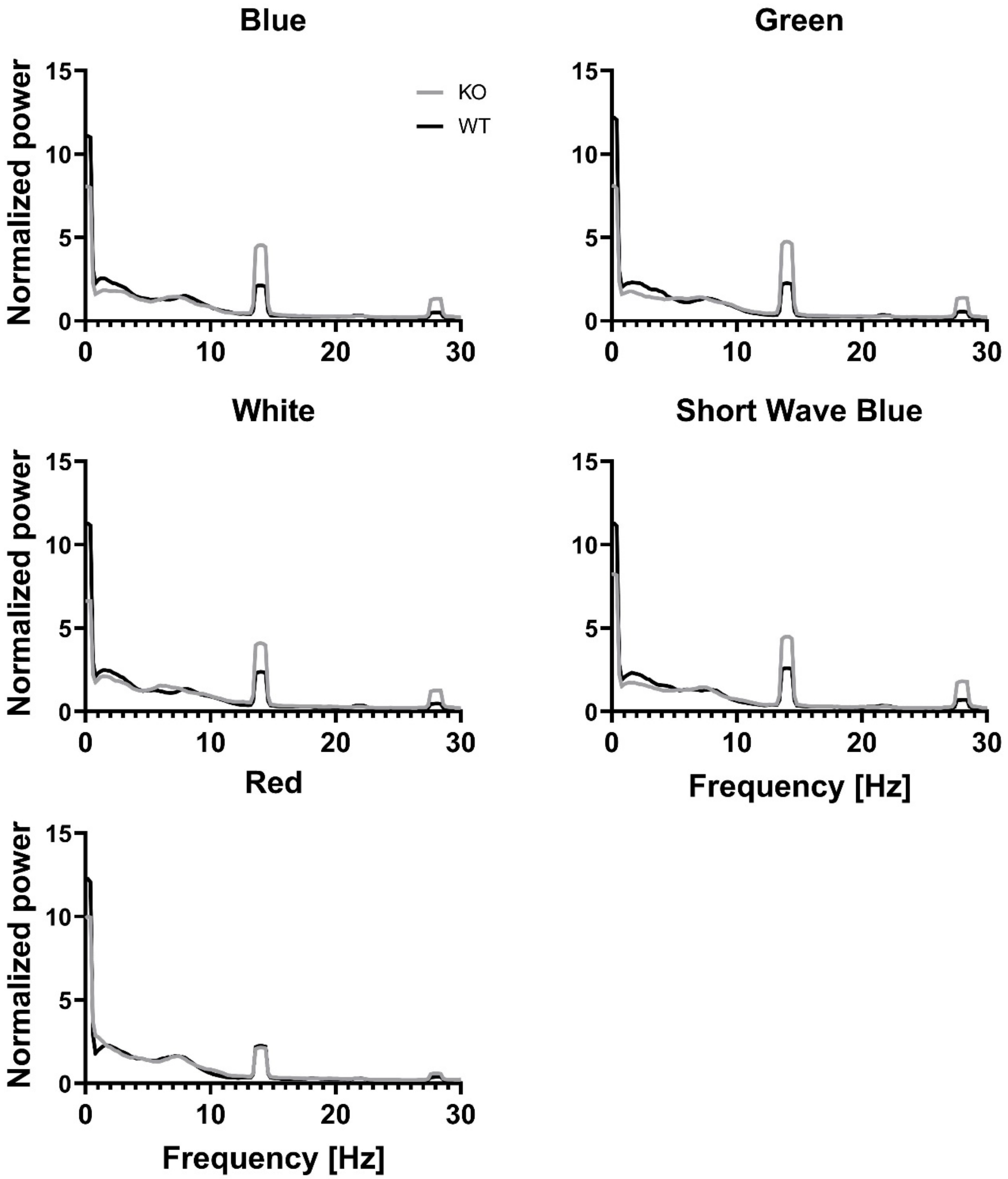

Figure S 4 Steady-state response from the Superior Colliculus. The delta showed a significant interaction of genotype and colour F(4,108)=3.33, p=0.130. The post-hoc showed that for the KO animal the red condition was significantly different from the other colour conditions. The 1st harmonic showed a significant interaction between colour and genotype F(4,108)=10.1, p<0.0001. The post-hoc showed a significant red condition for the KO animals. The KO and WT differed significantly in the green (z=-3.91, p=0.003) and the blue (z=-3.75, p=0.005) condition. The 2nd harmonic showed a significant interaction between colour and genotype F(4,108)=9.16, p<0.0001. The post-hoc revealed that the SWB and red conditions differed from all other conditions for the KO animals. The WT and KO animals differed from each other in the blue (z=-4.82, p<0.001), green (z=-4.85, p<0.001), SWB (z=-6.44, p<0.001) and white (z=-4.63, p<0.001) conditions.

Table 2S SSVEP from superior colliculus. Normalized power and frequency as mean (standard deviation). SWB: short-wave blue, WT: wildtype, KO: knock-out.

Superior Colliculus		WT	N=14	KO	N=15
Peak	Colour	power	frequency	power	frequency
Delta	SWB	3.044 (0.658)	1.8 (1.014)	2.283 (0.307)	1.307 (0.767)
	Blue	3.224 (0.628)	1.171 (0.35)	2.438 (0.651)	1.493 (0.851)
	Green	3.445 (1.112)	1.6 (0.928)	2.436 (0.754)	1.427 (0.663)
	White	3.217 (1.085)	1.443 (0.643)	2.395 (0.747)	1.507 (0.658)
	Red	3.137 (1.067)	1.586 (0.965)	3.833 (2.357)	1.067 (0.258)
1. Harmonic	SWB	2.616 (1.697)	14.371 (0.19)	4.502 (1.752)	14.427 (0.128)
	Blue	2.157 (1.376)	14.214 (0.589)	4.546 (2.058)	14.44 (0.083)
	Green	2.266 (1.271)	14.443 (0.14)	4.76 (1.977)	14.347 (0.092)
	White	2.385 (1.706)	14.443 (0.224)	4.123 (2.106)	14.4 (0.151)
	Red	2.265 (1.664)	14.414 (0.183)	2.173 (1.241)	14.28 (0.56)
2. Harmonic	SWB	0.713 (0.327)	28.557 (0.25)	1.82 (0.676)	28.467 (0.145)
	Blue	0.505 (0.319)	28.443 (0.21)	1.332 (0.647)	28.52 (0.166)
	Green	0.553 (0.334)	28.357 (0.224)	1.386 (0.565)	28.453 (0.092)
	White	0.482 (0.263)	28.6 (0.422)	1.277 (0.595)	28.44 (0.188)
	Red	0.402 (0.157)	28.643 (0.438)	0.606 (0.354)	28.387 (0.207)

Table 3 S F-values from the 2-way ANOVA of colour and genotype of SSVEP.

F-values for 2-way ANOVA			*colour*	*genotype*	*colour*genotype*
Visual cortex	Normalized power	Delta			F(4,108)=3.47, p=0.0105
		Alpha			F(4,108)=3.4, p=0.0117
		1.harm			F(4,108)=10.6, p<.0001
		2.harm	F(4,108)=11.5, p<.0001		
	Peak frequency	Delta			
		Alpha			
		1.harm			
		2.harm			
Superior colliculus	Normalized power	Delta			F(4,108)=3.33, p=0.0130
		1.harm			F(4,108)=10.1, p<.0001
		2.harm		F(4,108)=9.16, p<.0001	
	Peak frequency	Delta			
		1.harm			
		2.harm			

Table 4 S Mean (SD) of SSVEP data from the PFE360 test.

Visual Cortex		*WT*	*N=14*	*KO*	*N=15*
Peak Delta	Drug	power	frequency	power	frequency
	Vehicle	2.921 (1.402)	1.286 (0.39)	2.082 (0.947)	1.418 (0.648)
	PFE360	2.833 (1.759)	1.471 (0.65)	1.951 (1.106)	1.596 (0.738)
Alpha	Vehicle	3.109 (1.188)	7.981 (0.98)	3.922 (1.088)	7.422 (0.704)
	PFE360	2.943 (0.96)	7.252 (1.266	4.312 (1.052)	6.911 (0.79)
1st Harmonic	Vehicle	2.056 (0.948)	14.395 (0.121)	1.702 (0.596)	14.324 (0.305)
	PFE360	2.2 (0.965)	14.381 (0.106)	1.421 (0.506)	14.187 (0.523)
2nd Harmonic	Vehicle	1.236 (0.695)	28.414 (0.162)	0.867 (0.395)	28.467 (0.154)
	PFE360	1.076 (0.57)	28.429 (0.113)	0.886 (0.487)	28.427 (0.163)
Superior Colliculus		WT	N=14	KO	N=15
Peak Delta	Drug	power	frequency	power	frequency
	Vehicle	2.917 (0.868)	1.548 (0.839)	2.61 (2.02)	1.809 (1.118)
	PFE360	3.281 (2.154)	2.01 (1.142)	2.471 (1.399)	2.404 (1.224)
1st Harmonic	Vehicle	2.119 (0.921)	14.252 (0.492)	3.733 (1.874)	14.373 (0.157)
	PFE360	1.964 (0.85)	14.286 (0.496)	3.364 (1.791)	14.404 (0.157)
2nd Harmonic	Vehicle	0.506 (0.27)	28.49 (0.29)	1.271 (0.593)	28.569 (0.165)
	PFE360	0.529 (0.278)	28.357 (0.386)	1.411 (0.814)	28.547 (0.178)

Pilot with washout of PFE360

A small pilot with the washout of PFE360 was tested. However, the magnitude of change caused by PFE360 is very small. Further, for practical reasons these were only results from four WT animals, some of the variation means that there are fewer detectable differences when comparing any timepoint to the baseline. However, most of the effects detected in the cross-over study were reproduced with this smaller setup.

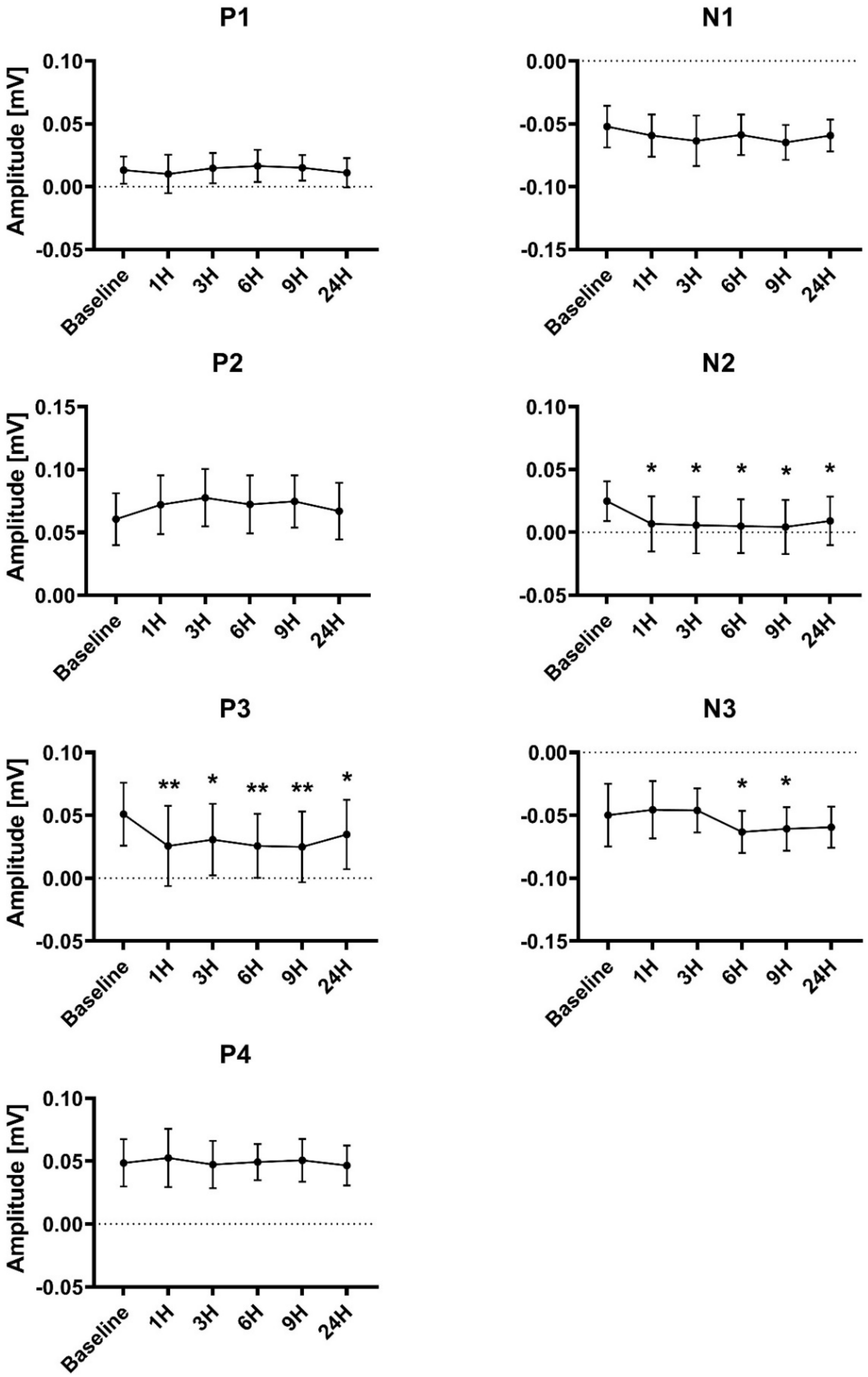

*Figure S 5 Washout of PFE360 amplitude of VEP recorded in the visual cortex. Asterisks refer to results from the post-hoc tests: * p<0.05, ** p<0.01.*

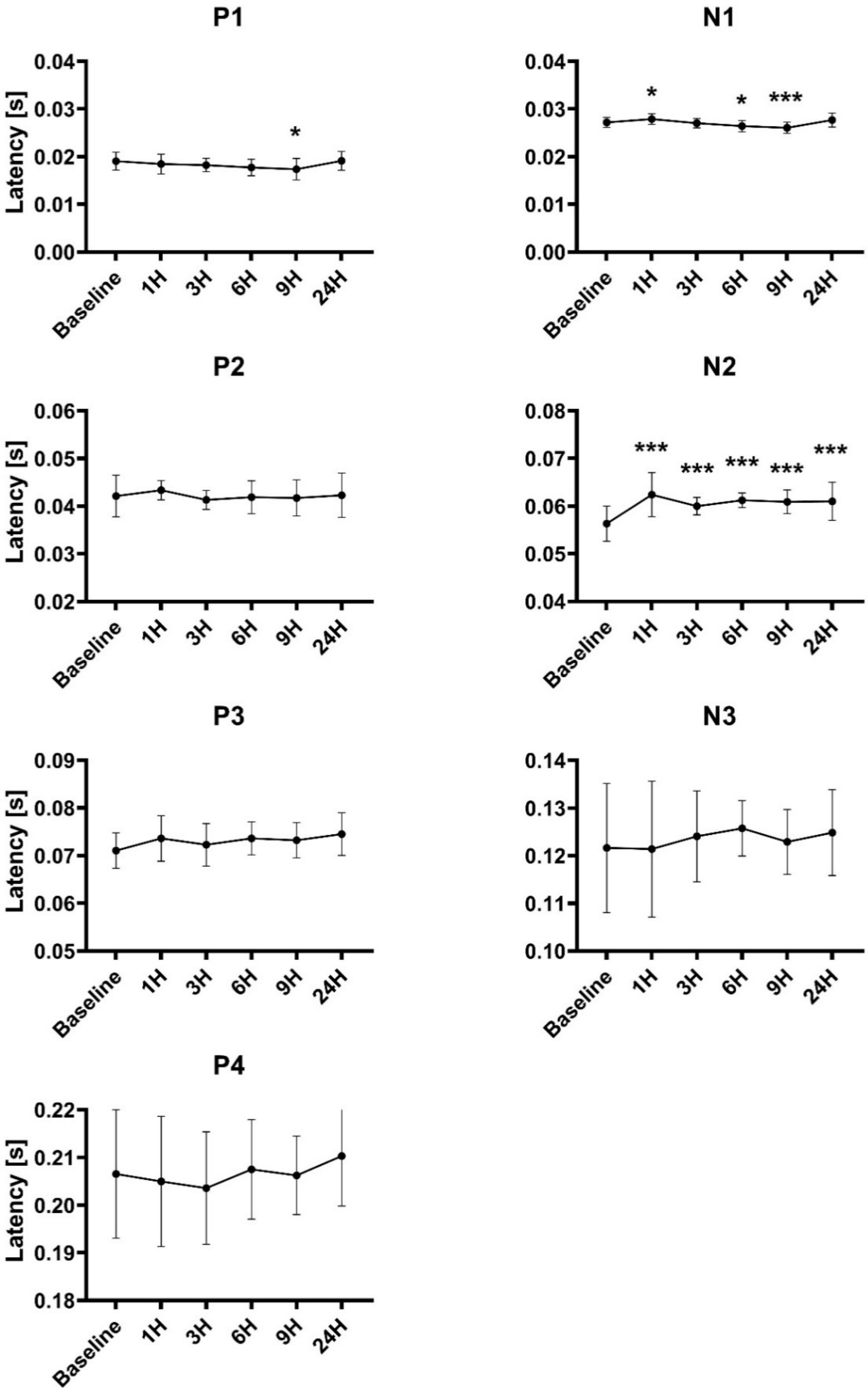

*Figure S 6 Washout of PFE360 latency of VEP recorded in the visual cortex. Asterisks refer to results from the post-hoc tests: * p<0.05, ** p<0.01, *** p<0.001.*

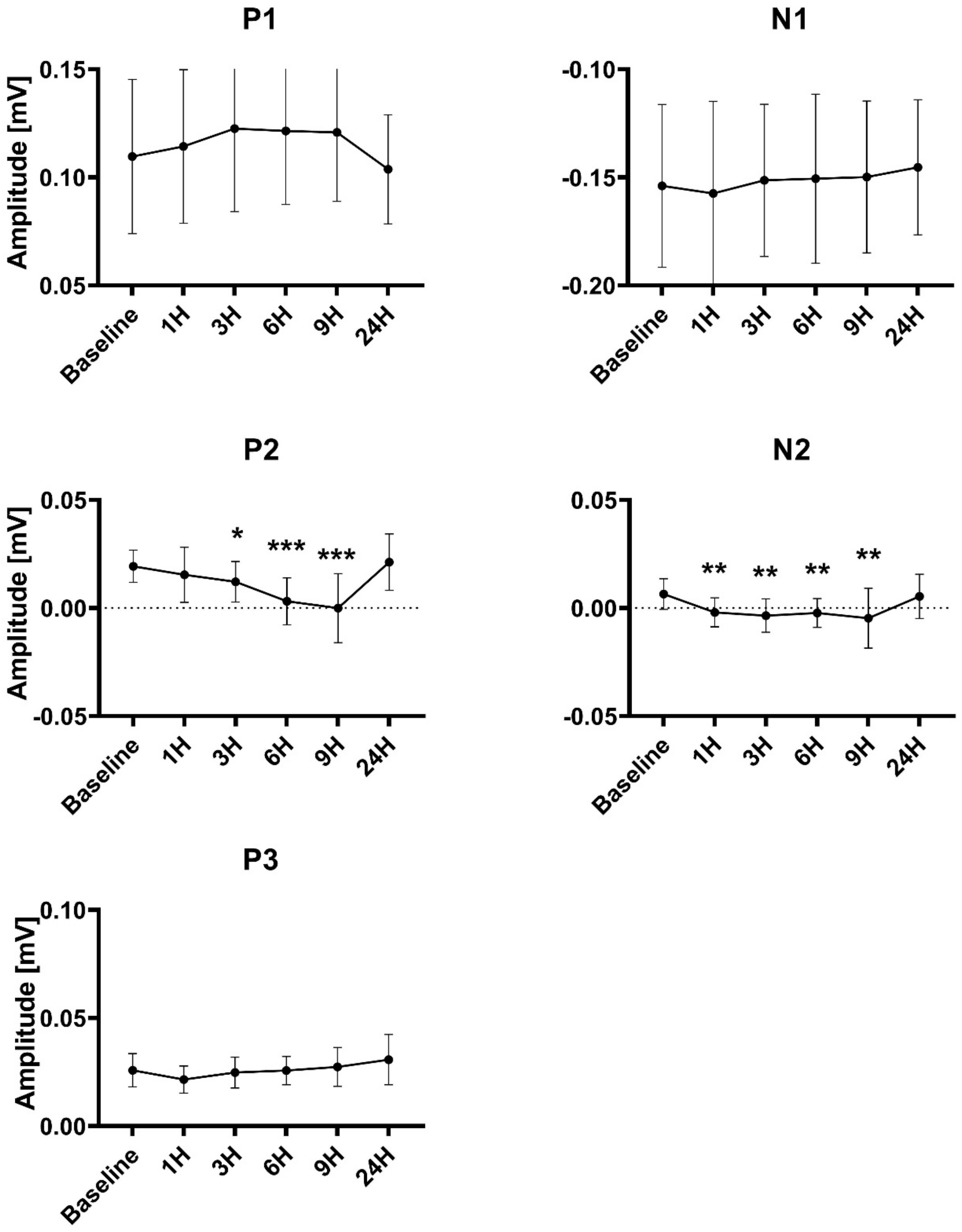

*Figure S 7 Washout of PFE360 amplitude of VEP recorded in the superior colliculus. Asterisks refer to results from the post-hoc tests: * p<0.05, ** p<0.01, *** p<0.001.*

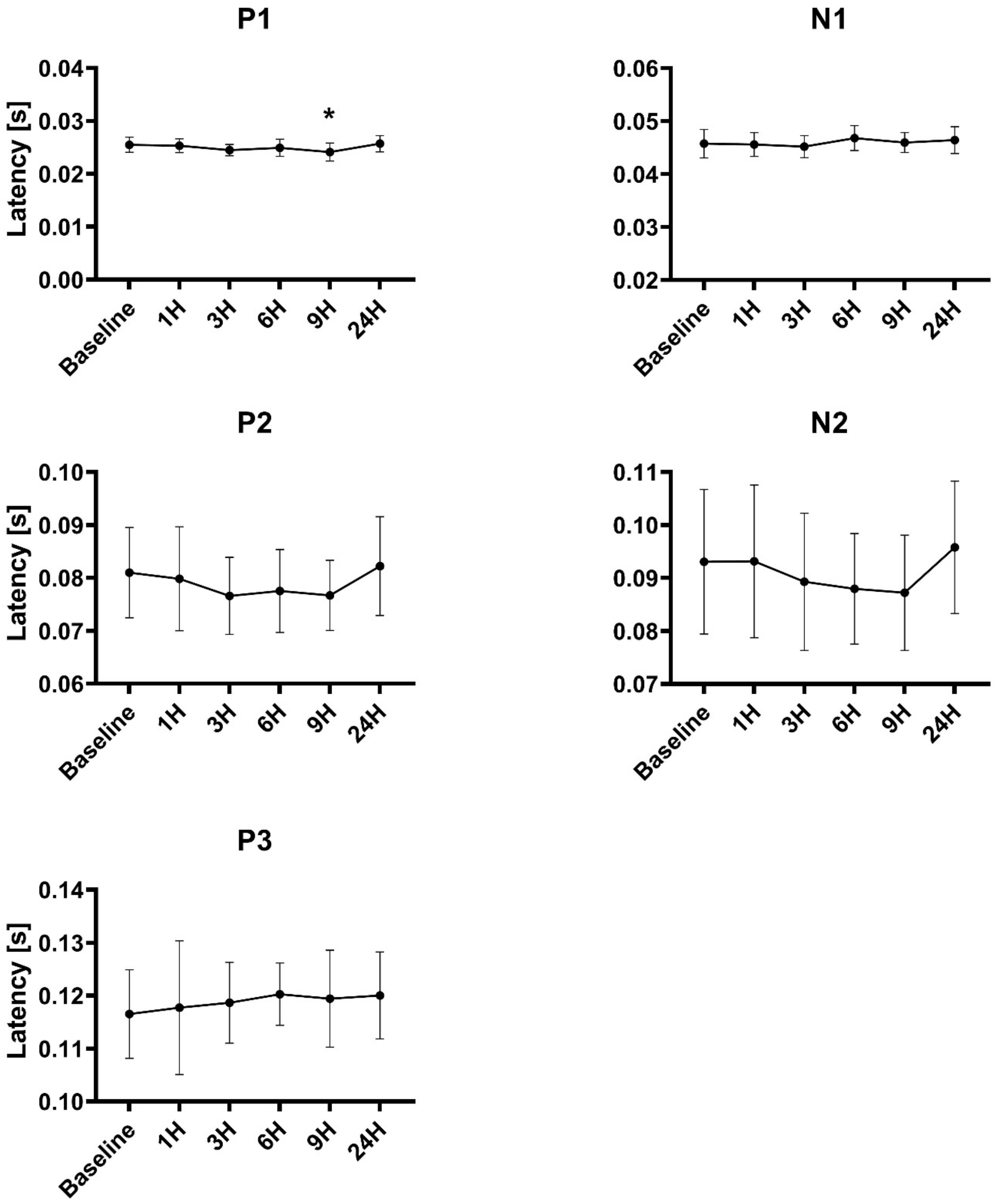

*Figure S 8 Washout of PFE360 amplitude of VEP recorded in the superior colliculus. Asterisks refer to results from the post-hoc tests: * p<0.05.*
